# Absorption of foliar-applied Zn fertilizers by trichomes in soybean and tomato

**DOI:** 10.1093/jxb/ery085

**Published:** 2018-03-03

**Authors:** Cui Li, Peng Wang, Enzo Lombi, Miaomiao Cheng, Caixian Tang, Daryl L Howard, Neal W Menzies, Peter M Kopittke

**Affiliations:** 1 The University of Queensland, School of Agriculture and Food Sciences, St Lucia, Queensland, Australia; 2 Nanjing Agricultural University, College of Resources and Environmental Sciences, Nanjing, China; 3 University of South Australia, Future Industries Institute, Mawson Lakes, South Australia, Australia; 4 La Trobe University, Centre for AgriBioscience, Bundoora, Victoria, Australia; 5 ANSTO, Australian Synchrotron, Clayton, Victoria, Australia

**Keywords:** Foliar absorption, foliar fertilizer, trichomes, Zn, soybean, tomato, nanoparticles

## Abstract

The present study investigated the role of trichomes in absorption of foliar-applied zinc fertilizers in soybean and tomato. Using synchrotron-based X-ray fluorescence microscopy for *in situ* analyses of hydrated leaves, we found that upon foliar application of ZnSO_4_, Zn accumulated within 15 min in some non-glandular trichomes in soybean, but not in tomato. However, analyses of cross-sections of soybean leaves did not show any marked accumulation of Zn in tissues surrounding trichomes. Furthermore, when near-isogenic lines of soybean differing 10-fold in trichome density were used to compare Zn absorption, it was found that foliar Zn absorption was not related to trichome density. Therefore, it is suggested that trichomes are not part of the primary pathway through which foliar-applied Zn moves across the leaf surface in soybean and tomato. However, this does not preclude trichomes being important in other plant species, as they are known to be highly diverse. We also compared the absorption of Zn when supplied as either ZnSO_4_, nano-ZnO, or bulk-ZnO, and found that absorption from ZnSO_4_ was about 10-fold higher than from nano- and bulk-ZnO, suggesting that it was mainly absorbed as soluble Zn. This study improves our understanding of the absorption of foliar-applied nutrients.

## Teaser

Leaf trichomes are not part of the primary pathway through which foliar-applied Zn fertilizer moves across the leaf surface in soybean and tomato.

## Introduction

Zinc (Zn) is an essential micronutrient for the nutrition of both plants and humans. However, it has been estimated that 49% of the world’s agriculturally important soils have inadequate levels of Zn and that one-third of the world’s human population suffers from Zn deficiency (Sillanpää, 1990; Cakmak *et al.*, 2010). Foliar fertilization is potentially an efficient approach for the correction of Zn deficiency in crops (Zhang *et al.*, 2018; Jaksomsak *et al.*, 2018). The use of foliar fertilizers is particularly useful for those soils that limit the uptake of Zn by the roots due to chemical constraints, such as alkaline soils in which Zn availability is low due to precipitation reactions (Cakmak, 2008; Cakmak *et al.*, 2010). However, much remains unknown regarding the underlying processes whereby foliar-applied nutrients move across the leaf surface into the underlying plant tissue (Fernández *et al.*, 2017; Li *et al.*, 2017). This poor understanding of the physiological processes involved in the absorption of foliar-applied nutrients hinders efforts to increase the effectiveness of foliar fertilizers (Fernández *et al.*, 2013). Of particular interest, it is unclear how nutrients move across the hydrophobic cuticle covering the leaf surfaces (Riederer and Schreiber, 2001; Fernández and Eichert, 2009; Fernández *et al.*, 2017). For example, the importance of the stomata, cuticle, and trichomes in foliar nutrient absorption remains unclear (Schönherr, 2006; Fernández *et al.*, 2013).

In the present study, we focus on the role of trichomes for the foliar-absorption of Zn. Trichomes are generally found on both the abaxial and adaxial surfaces of leaves, and can be found at high densities in many plant species. For example, glandular trichomes have been reported on leaves of tomato (*Solanum lycopersicum*) at a density of 11100 cm^−2^ (Maluf *et al.*, 2007). Trichomes are appendages that originate from epidermal cells and develop outwards on the surface of various plant organs (Werker, 2000). Depending on the species, the growth environment, and the organs where they are located, trichomes vary considerably in structure, morphology, size, and function (Werker, 2000; Ohrui *et al.*, 2007). They can be broadly classified as being either glandular or non-glandular, with the major distinction being that glandular trichomes can contain or excrete various chemical compounds (Tian *et al.*, 2012). The most well-known function of trichomes is for the protection of plants from herbivores, both through a physical interference and through the excretion of toxic or repellent compounds (Levin, 1973; Tian *et al.*, 2012; Aschenbrenner *et al.*, 2013; Stratmann and Bequette, 2016). Although it has been reported for several plant species that trichome bases are cutinized (Fahn, 1986; Mahlberg and Kim, 1991; Ascensão *et al.*, 1999; Huang *et al.*, 2008; Fernández *et al.*, 2011, 2014; Wu *et al.*, 2011; Guo *et al.*, 2013), it has also been reported that they can absorb water in some species; for example, the trichomes of a cactus (*Opuntia microdasys*) are involved in its fog collection system (Ju *et al.*, 2012) and trichomes of *Phlomis fruticosa* can absorb water under drought conditions (Grammatikopoulos and Manetas, 1994). In contrast, Burrows *et al.* (2013) reported that the stellate trichomes of *Solanum elaeagnifolium* were water-repellant and were likely to be barriers for absorption of foliar-applied chemicals. In addition, it has been found that depending upon their density, structure, and chemical composition, trichomes can influence leaf surface wettability and droplet-retention ability. Specifically, higher trichome densities generally lead to lower wettability but greater droplet retention (Brewer *et al.*, 1991; Grammatikopoulos and Manetas, 1994; Fernández *et al.*, 2014), and it was found that some trichomes have a higher water sorption capacity than the cuticle (Fernández *et al.*, 2011). However, much uncertainty remains regarding the role (if any) of trichomes in the absorption of foliar-applied nutrients.

Given that trichomes are the outermost aerial structure of leaves, it is important to elucidate their role in the absorption of foliar-applied nutrients—and this formed the aim of the present study. Using soybean (*Glycine max*) and tomato, we first examined the morphology of trichomes on the leaf surface using scanning electron microscopy (SEM) and light microscopy. Next, we used synchrotron-based X-ray fluorescence microscopy (µ-XRF) for the *in situ* examination of fresh (hydrated) leaves to obtain laterally resolved elemental maps showing the distribution of foliar-applied Zn, and the spatial distribution within the soybean leaf was also examined. Finally, for soybean, we used four near-isogenic lines (NILs) that varied 10-fold in trichome density to compare foliar Zn absorption by examining bulk leaf Zn concentrations. For this study, we also compared three types of Zn fertilizers, namely ZnSO_4_, nano-ZnO, and bulk-ZnO (all applied at 1000 mg l^−1^). This is important given that there is increasing interest in the potential use of nanoparticles as foliar fertilizers (DeRosa *et al.*, 2010; Nair *et al.*, 2010; Khot *et al.*, 2012; Wang *et al.*, 2016). The results provide important information regarding the movement of nutrients across the leaf surface, and have the potential to assist in improving the efficiency of Zn foliar fertilizers.

## Materials and methods

### Plant growth and assessment of leaf trichomes

Plants were grown at The University of Queensland (St Lucia, Australia), at 25 °C, with artificial light provided by high-pressure sodium lamps (photon flux density of 1500 μmol m^–2^ s^–1^) for 12 h d^−1^. Seeds of soybean (cv. Bunya) and tomato (cv. Red Luck) were placed in rolled paper towels suspended vertically in tap water for either 3 d (soybean) or 4 d (tomato). Seedlings were then transferred to 11-l black buckets, with the lids having four holes in which the seedlings were suspended using shade cloth. Each bucket had eight plants (two plants per hole). The buckets were filled with a nutrient solution that contained (in µM): 910 N (94% NO_3_^−^ and 6% NH_4_^+^), 475 K, 20 P, 1126 Ca, 227 Mg, 1251 Cl, 556 S, 25 Fe(III)EDTA, 3 B, 0.5 Mn, 0.5 Zn, 0.2 Cu, and 0.01 Mo (Blamey *et al.*, 2015). The nutrient solutions were changed after 1 week, and then every 4 d. In addition, to replace P being taken up from the solution, 5 ml of 44 mM KH_2_PO_4_ was added every second day once the plants had been growing in the solution for 10 d.

The youngest fully expanded leaves (YFELs) were used to examine trichome morphology after 14 d (soybean) and 18 d (tomato), as outlined by Li *et al.* (2017). Briefly, the fresh YFELs were cut into small segments (2 × 4 mm) and then fixed in 3% glutaraldehyde in 0.1 M sodium cacodylate with a microwave processing, followed by 1% OsO_4_ in 0.1 M sodium cacodylate. Next, samples were dehydrated using an ethanol gradient with a Pelco BioWave (Ted Pella Inc., California, USA), dried using a critical-point dryer (Autosamdri-815 CPD, USA), and coated with Au. The trichomes were analysed using a SEM (JEOL NEOSCOPE, Japan) at 10 kV accelerating voltage.

To examine trichome structure, cross-sections of the leaves were cut and analysed using light microscopy as described by Li *et al.* (2017). Briefly, the YFELs were cut into 1 × 4-mm segments and fixed overnight in 3% glutaraldehyde with 0.1 M sodium cacodylate at 4 °C. Using 0.1 M sodium cacodylate, the samples were rinsed before being post-fixed in 1% OsO_4_ in 0.1 M sodium cacodylate for 4 h, and then dehydrated in a graded ethanol series. The samples were infiltrated with a series of graded Epon mixtures (Epon in ethanol) and then embedded in 100% Epon blocks and polymerized at 60 °C overnight. An ultramicrotome (LEICA EM UC6) was used to cut the sections (1 µm) before staining using Toluidine blue (0.5% in 1% borax) (O’Brien, 1995).

### Examining the distribution of Zn using µ-XRF

Soybean and tomato were grown at La Trobe University (Bundoora, Australia) before being transported to the Australian Synchrotron (Clayton, Australia). Plants were grown as described above. After growth in the nutrient solution for 14 d (soybean) and 18 d (tomato), two droplets (5 µl each) of 1000 mg l^−1^ of Zn solution (supplied as ZnSO_4_.7H_2_O, 15.4 mM, pH 5.2) were applied to the adaxial surface of the YFELs. The droplets also contained 0.05% Tween 20 to decrease surface tension (Reuveni *et al.*, 1994). The leaves were sealed in Petri dishes that had a hole to allow for the petiole, with the leaves still attached to the plants. The Petri dishes also contained moist filter paper, with the droplets left on the leaves for 0.25, 0.5, 1, 3, or 6 h. During this time, the droplets did not dry out but remained as a liquid, with relative humidity (RH) inside the sealed Petri dish increasing rapidly to 98% (temperature of 30 °C). At the end of the time period, the droplets were blotted dry with filter paper, and the leaves were excised and rinsed sequentially using 2% HNO_3_, 3% ethanol, and deionized water (Du *et al.*, 2015).

The thoroughly rinsed leaves were blotted dry and analysed using µ-XRF at the XFM beamline at the Australian Synchrotron. The leaves were mounted tightly between two pieces of 4-µm thick Ultralene film, which formed a seal around the leaf to limit dehydration. The time between excision of the leaf and commencement of the µ-XRF analysis was <5 min. Basic information of the XFM beamline is given by Kopittke *et al.* (2011) and Paterson *et al.* (2011). Briefly, X-rays were selected by a Si (111) monochromator and focused (approx. 2 × 2 µm) on the specimen by a pair of Kirkpatrick–Baez mirrors. A 384-element Maia detector system in a backscatter position (180°) at an excitation energy of 12900 eV was used to collect the X-ray fluorescence emitted by the specimen. For each specimen, a ‘survey scan’ and a ‘detailed scan’ were performed. The survey scan was a comparatively quick scan that was used to allow for the selection of an area of interest; the step size (i.e. virtual pixel size) was 30 µm with a horizontal stage velocity of 4 mm s^−1^, resulting in a pixel transit time of 7.5 ms. For the subsequent detailed scan, the step size was 1 µm with a horizontal stage velocity of 1 mm s^−1^, resulting in a pixel transit time of 1 ms. Although it varied, the survey scans were often approx. 10 × 6 mm in size (resulting in a scan duration of ~10 min) and the detailed scans were often approx. 4 × 5 mm in size (resulting in a scan duration of ~75 min). The full X-ray fluorescence spectra were analysed using the CSIRO Dynamic Analysis method in GeoPIXE (http://www.nmp.csiro.au/dynamic.html) (Ryan and Jamieson, 1993; Ryan, 2000).

For soybean, cross-sections of the leaf tissues were also prepared for examining the distribution of Zn. The samples were prepared at The University of Queensland, with soybean plants grown for 14 d as described above. At 14 d, a 40-µl droplet of 1000 mg l^−1^ Zn (supplied as ZnSO_4_.7H_2_O with 0.05% Tween 20) was applied on the adaxial leaf surface before being incubated in Petri dishes for either 0 (control), 0.25, 1, or 6 h. We applied a larger volume in this experiment (40 µl instead of 5 µl) in order to ensure that the resulting cross-section that was cut came from an area entirely below the droplet. After incubation, the area below where the Zn had been applied (or the corresponding position on the control leaf) was excised after being rinsed, and the leaf segments were embedded in 4% agar solution at ~30 °C. After the agar solidified, the block was attached to the sample holder of a vibratome (Leica VT 1000s) using superglue, with 150-µm thick cross-sections obtained without use of a buffering solution. Finally, the sections were sealed in Ultralene film with XRF Sample cups (SC-8047, Premier Lab Supply), small holes were made in the film, and the samples were freeze-dried.

At the XFM beamline, the sections were carefully removed and placed between two sheets of Ultralene film (4 µm) and stretched over a Perspex frame, which was magnetically mounted on the motion stage. A Vortex detector system at a 90° angle and an excitation energy of 15800 eV was used to collect the X-ray fluorescence emitted by the specimen. Again, a ‘survey scan’ was used for each sample to first identify the area of interest before obtaining a higher-resolution image (‘detailed scan’). For the ‘survey scan’, the step size was 50 µm with a horizontal stage velocity of 1 mm s^−1^, resulting in a pixel transit time of 50 ms. For the subsequent ‘detailed scan’, the step size was 2 µm with a horizontal stage velocity of 0.6 mm s^−1^, resulting in a pixel transit time of 3.33 ms. The survey scans generally took ~3 min, and the detailed scans generally took ~30 min.

### Zinc absorption using soybean mutants

Four NILs of soybean (cv. Clark) were obtained from the USDA Soybean Germplasm Collection, Department of Crop Sciences, USDA/ARS University of Illinois, Urbana IL, USA. The NILs were wild-type Clark (L58-231) (CS), sparse trichome (L63-2999) (Ps), dense trichome (L62-1686) (Pd1), and glabrous (L62-1385) (P1). Plants were grown at The University of Queensland (St Lucia, Australia) as described above, except that plants were grown in 5-l buckets with six plants in each bucket. Each of these buckets formed a single experimental unit, with six replicates per genotype.

For this experiment we also compared three forms of Zn, with it being supplied as ZnSO_4_.7H_2_O, nano-ZnO (~30 nm diameter), and its homologous bulk-ZnO (~200–400 nm diameter) (Supplementary Fig. S1, Table S1 at *JXB* online). The two ZnO forms (nano and bulk) were analysed using dynamic light scattering (DLS) using a zeta potential / particle sizer NICOMP 380 ZLS at a concentration of 333 mg l^-1^. For this experiment, all three forms were used at a total Zn concentration of 1000 mg l^−1^. Although ZnSO_4_.7H_2_O is highly soluble, nano-ZnO and bulk-ZnO are of only sparingly solubility, and hence although the total Zn concentration of the suspensions of these two compounds was 1000 mg l^−1^, the concentration of soluble Zn would be lower than this value. Therefore, we measured the concentration of soluble Zn in suspensions of 1000 mg l^−1^ nano- and bulk-ZnO. To do this, suspensions were mixed for 15 min in an ultrasonic bath before being filtered using centrifugal filter units (3 kDa, Millipore, Sigma). The Zn concentrations were measured in the filtered solutions using inductively coupled plasma mass spectrometry (ICP-MS). For both the nano- and bulk-ZnO suspensions, the Zn concentration was 3 mg l^−1^ (46 µM). Therefore, to help in comparing the 1000 mg l^−1^ nano- and bulk-ZnO suspensions with the ZnSO_4_.7H_2_O solution, we also included a treatment with 3 mg l^−1^ ZnSO_4_.7H_2_O. All solutions contained 0.05% Tween 20. In addition, we examined two application times, 1 and 6 h. Thus, the experiment consisted of a total of 32 treatments, with four NILs of soybean differing in trichome density, two application times (1 and 6 h), and four Zn treatments: 1000 mg l^−1^ ZnSO_4_.7H_2_O, 3 mg l^−1^ ZnSO_4_.7H_2_O (pH 5.2), 1000 mg l^−1^ nano-ZnO (pH 7.1), and 1000 mg l^−1^ bulk-ZnO (pH 7.0). Each treatment had six replicates, yielding a total of 192 experimental units.

After growth for 2 weeks, 30 droplets (5 μl each) of the appropriate Zn fertilizer were applied to half of the adaxial surface of the YFELs, with 30 droplets (5 μl each) of deionized water (with 0.05% Tween 20) applied to the other half of the leaf (Vu *et al.*, 2013). Following application of the droplets, the leaves were sealed inside Petri dishes with moistened filter paper, with the lights remaining switched on during the incubation period. As noted above, RH inside the sealed Petri dishes increased rapidly to 98%, and the droplets remained as a liquid during the experimental period (i.e. they did not dry out). After the appropriate application time (1 or 6 h), the leaves were cut in half along the mid-vein, and rinsed separately using 2% HNO_3_, 3% ethanol, and deionized water. The leaf tissues were oven-dried (65 °C), digested using a 5:1 mixture of nitric acid and perchloric acid, and analysed using ICP-MS. Blanks and reference materials were included to ensure accuracy. The data were calculated as Zn absorption (μg per half leaf) = (elemental concentration in the treated half – elemental concentration in the control half) × dry weight of the treated half of the leaf.

For analyses of the trichome and stomatal densities, each of the four NILs was grown for 2 weeks before examination using the SEM as described above, with six replicates examined. Finally, as it has been proposed that trichomes may alter leaf surface wettability (Fernández *et al.*, 2014), and so we measured the contact angle of 5-µl droplets for 1000 mg l^−1^ ZnSO_4_.7H_2_O, 1000 mg l^−1^ nano-ZnO, and 1000 mg l^−1^ bulk-ZnO (all with 0.05% Tween 20) for the four NILs using the drop shape method (Woodward, 1999).

### Statistical analyses

Data were analysed using IBM SPSS version 24 and GenStat version 18. Comparisons between means were made using the one-way analysis of variance least significant difference (LSD) at 5%.

## Results

### Types and morphology of trichomes on leaves of soybean and tomato

**Fig. 1.**
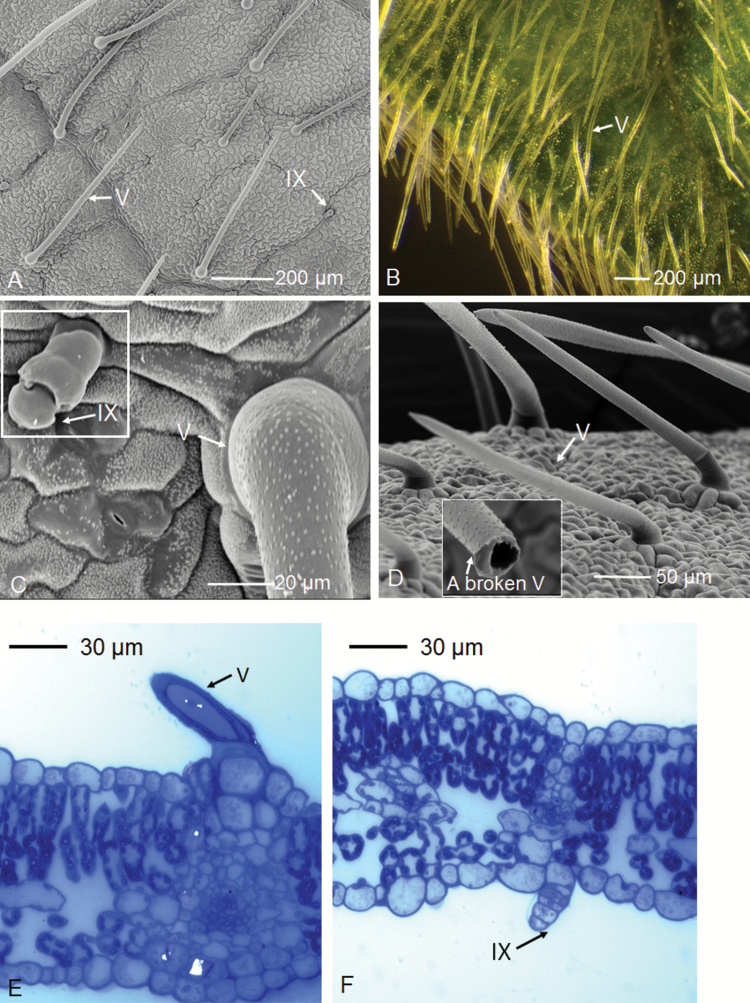
Trichomes on the leaf surface of soybean. Type V and Type IX are indicated by ‘V’ and ‘IX’, respectively. Leaves were analysed using light microscopy (B, E, F) and SEM (A, C, D). (A–D) Trichomes on the adaxial leaf surface, with the Type V trichome in (D) broken and showing a cross-section. (E, F) Leaf cross-sections stained with Toluidine blue.

For soybean, two types of trichomes were found (Fig. 1); a non-glandular trichome (Type V) and a glandular trichome (Type IX) (Franceschi and Giaquinta, 1983). The Type IX trichomes were ~0.04 mm in length and consisted of about four stalk cells with one small cell on the tip (Fig. 1A, C, F). In contrast, the Type V trichomes were hollow, consisting of a multicellular base and one long stalk cell up to 1 mm in length (Fig. 1A, B, D, E). The overall trichome density of the soybean adaxial leaf surface was 1300 cm^−2^, with 65% being non-glandular trichomes (Type V) (Fig. 1).

**Fig. 2.**
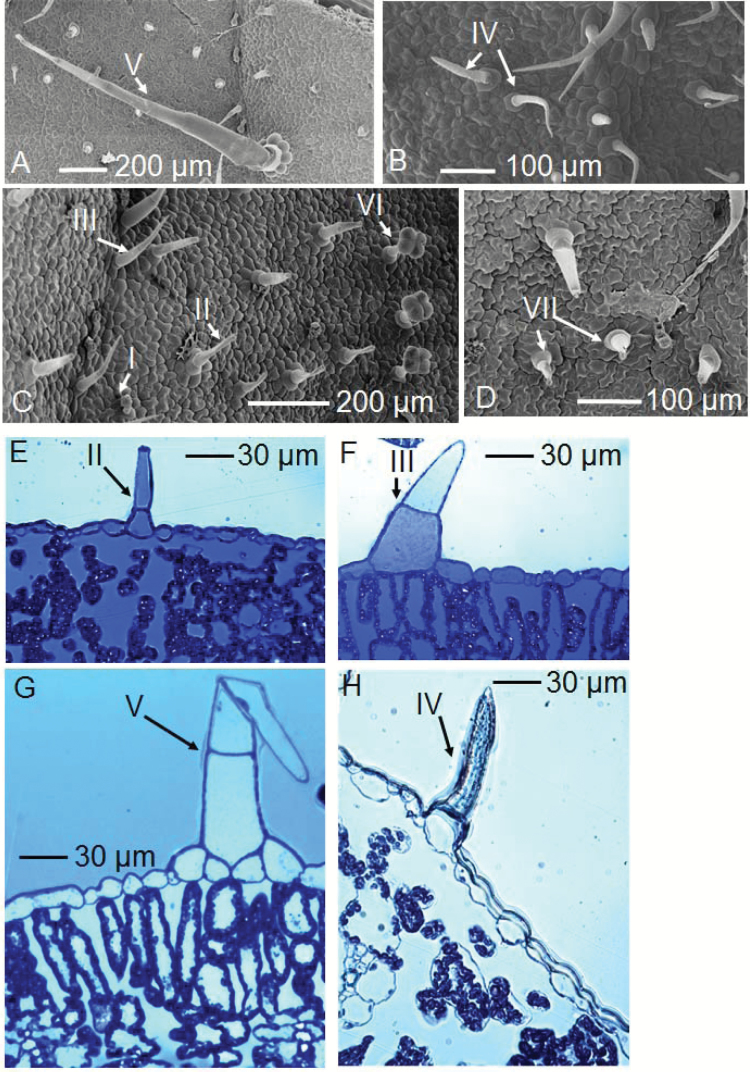
Trichomes on the leaf surface of tomato. The seven types are indicated with ‘I’–‘VII’. Leaves were analysed using light microscopy (E–H) and SEM (A–D). (A–D) Trichomes on the adaxial leaf surface. (E–H) Leaf cross-sections stained with Toluidine blue.

Tomato leaves had a total of seven types of trichomes, with four types of glandular trichomes (Types I, II, VI, and VII) and three types of non-glandular trichomes (Types III, IV, and V) (Fig. 2). The Type I trichomes were the smallest, consisting of a short stalk cell with a capitate round cell on the top (Fig. 2C). The Type II and VI trichomes were similar in length, with both having a unicellular base connected to a stalk cell, but the Type VI trichomes had a four-cell head (Fig. 2C–E). The Type III and IV trichomes were both non-glandular and were similar in shape, with the Type III having two stalk cells while the Type IV had only one long stalk cell (Fig. 2B, C, F, H). Type V trichomes were the largest observed on the tomato leaf, consisting of a multi-cellular base connected with a long stalk (up to >2 mm) (Fig. 2A, G). The Type VII trichomes were similar to Type II, but they had a small round cell on the trichome tip (Fig. 2D). Overall, the tomato adaxial leaf trichome density was 1800 cm^−2^, with ~48% of the trichomes being Type II, followed by ~24% of Type III and IV, then Types VI (~13%), I (~9%), V (~5%), and VII (<1%) (Fig. 2).

### Laterally resolved elemental maps showing the movement of Zn into the leaf tissues

**Fig. 3.**
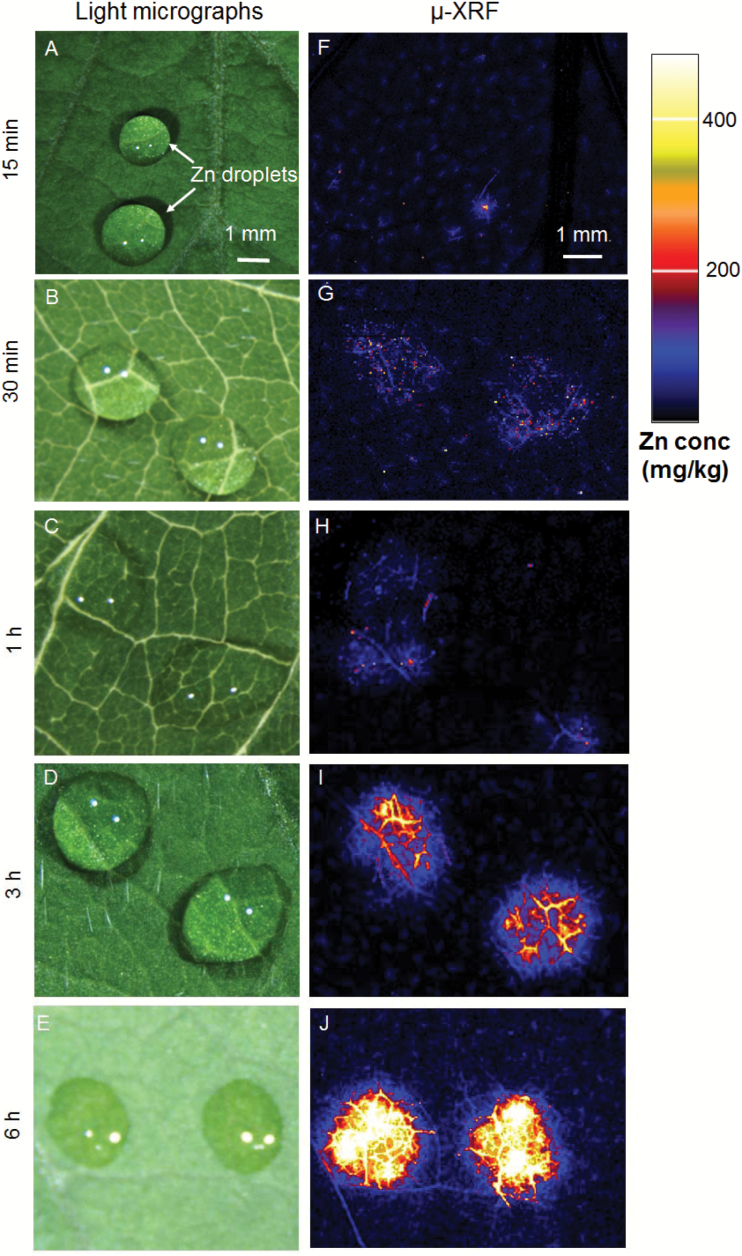
(A–E) Light micrographs showing droplets (ZnSO_4_) on the leaves of soybean. (F–J) Results from the µ-XRF survey scans showing the distribution of Zn. The images in (F–J) have the same color scale, with brighter colors corresponding to higher Zn concentrations, as shown in the key. Droplets were left on the leaf surface for between 15 min and 6 h, as indicated. The scale bar in (A) applies to (A–E), and the scale bar in (F) applies to (F–J).

The distribution of Zn (and other elements) was analysed *in situ* within fresh (hydrated) leaf tissues of soybean and tomato using synchrotron-based µ-XRF following exposure to 1000 mg l^−1^ of ZnSO_4_ for between 15 min and 6 h. Initially, rapid survey scans were used to allow for the selection of areas of interest, and these ) showed that within only 15 min the Zn had already moved across the leaf surface and was present in the leaf tissues below the Zn droplets for both species (Fig. 3 and Supplementary Fig. S2. The concentration of Zn within the leaf tissues gradually increased as the length of exposure increased from 15 min to 6 h. It was also observed that elevated levels of Zn were found almost exclusively in the tissues underlying the droplets. In other words, the movement of Zn away from the site of the application was limited.

**Fig. 4.**
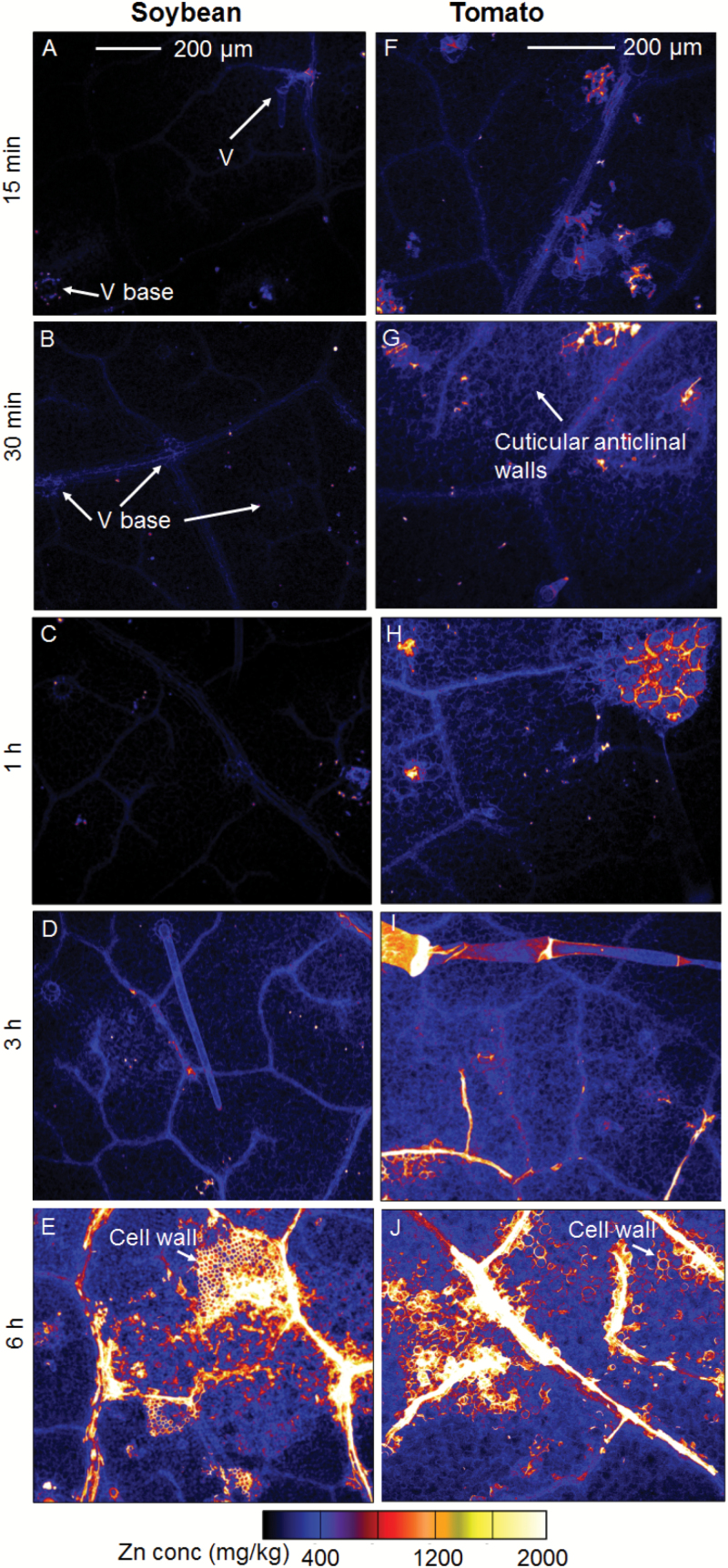
Detailed µ-XRF scans showing the distribution of Zn in hydrated (fresh) leaves of soybean (A–E) and tomato (F–J). All of the images were obtained from the area below a Zn droplet. The Zn droplets were applied to the leaf surface for between 15 min and 6 h, as indicated, and all the images have the same color scale, with brighter colors corresponding to higher Zn concentrations (as shown in the key). The scale bar in (A) applies to (A–E), the scale bar in (F) applies to (F–J).

Detailed scans were then used to more closely examine Zn distribution in the tissues directly underlying the application droplets. Soybean and tomato had similar Zn distribution patterns (Fig. 4), with Zn first observed to accumulate (i.e. within 15 min to 1 h) in the veins (Fig. 4A–C, F–H). For soybean, some Zn was also observed to accumulate in some of the basal cells of the non-glandular trichomes (Type V) (Fig. 4A–C). However, not all Type V trichomes below the droplets accumulated Zn. Assuming a trichome density of 1300 cm^−2^ and 65% being Type V (Fig. 1), it would be expected that there would be about seven Type V trichomes within the area shown in Fig. 4A–E, but only two (i.e. ~29% of the expected number) could be identified based upon the accumulation of Zn (Fig. 4A–D and Supplementary Fig. S3). For both species after exposure for 3 h, Zn had accumulated in almost all of the underlying veins as well as in the interveinal tissues (albeit at lower concentrations) (Fig. 4D, I). After 6 h, the extent of accumulation increased further, and within the interveinal tissues high concentrations of Zn were found to accumulate in the apoplast (Fig. 4E, J).

**Fig. 5.**
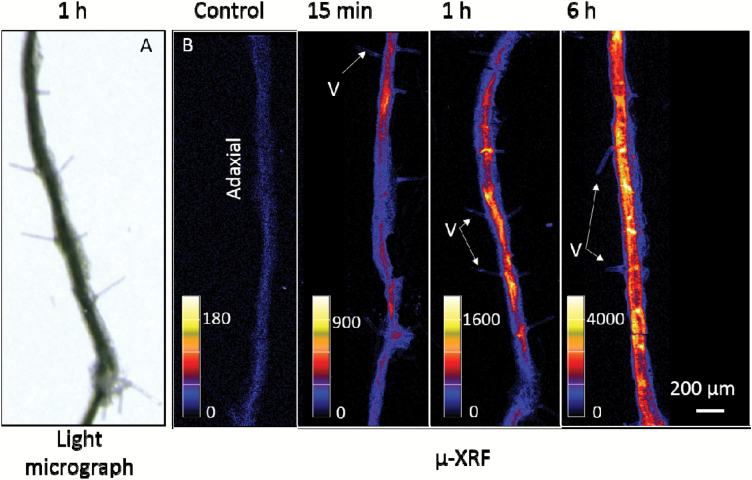
(A) A light micrograph of the cross-section of a soybean leaf that had Zn applied for 1 h before being scanned by µ-XRF. (B) µ-XRF scans showing the distribution of Zn in soybean leaves (150 µm leaf cross-sections). The sections were obtained from tissues underlying Zn droplets after 0 min (control), 15 min, 1 h, and 6 h of exposure. In each section, the adaxial side where the Zn had been applied is to the left. Brighter colors corresponded to higher Zn concentrations: note that the color is not comparable between the four samples, and individual scales are provided for each cross-section.

Finally, to further examine the role of the trichomes in foliar Zn absorption in soybean, we also used µ-XRF to examine cross-sections of the leaves cut from tissues underneath where droplets of Zn had been applied (the entire area of these cross-sections were obtained from underneath the droplets). Concentrations of Zn were found to have increased after only 15 min, and they continued increasing up to 6 h (Fig. 5). Interestingly, although Zn was found to accumulate in some of the non-glandular trichome bases in soybean, particularly in the first 3 h of foliar application (Fig. 4), analysis of the cross-sections by µ-XRF showed that the Zn concentration was not higher in the cells surrounding the base of these trichomes (Type V) (Fig. 5). This is probably because we could only observe a small number of trichomes in these cross-sections and only a limited number of them (~29% of the total Type V) were observed to have elevated levels of Zn (Fig. 4A–D and Supplementary Fig. S3). In addition, Zn was detected in the inner tissues of the leaves (presumably the mesophyll cells), particularly after 6 h application, implying that it had been transported into the leaf tissues after penetrating through the cuticle and epidermis (Fig. 5B). Given that the cross-sections were 150 µm thick (about eight cellular layers deep) (Fig. 1E,F), it was not possible to determine the subcellular distribution from them, and the apparent apoplastic accumulation observed from intact leaves (Fig. 4E) could not be confirmed from the cross-sections (Fig. 5).

### Foliar absorption in soybean NILs as related to trichome density

**Fig. 6.**
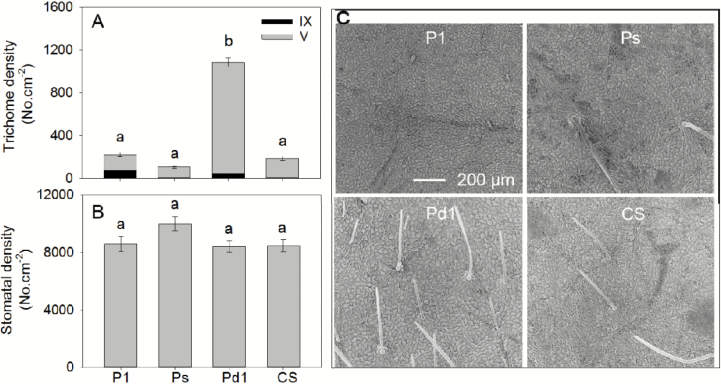
Densities of (A) trichomes and (B) stomata for leaves of four soybean near-isogenic lines (NILs): P1 (L62-1385), Ps (L63-2999), Pd1 (L62-1686), and CS (L58-231). In (A), ‘IX’ and ‘V’ refer to the two types of trichomes. Data in (A) and (B) are means (±SD) (*n*=6). Different letters indicate significant differences (*P*<0.05). (C) SEMs showing the trichomes in the four NILs. The scale bar for P1 applies to all four images.

Given that Zn was observed to accumulate rapidly in some trichomes of soybean (Fig. 4), we used four NILs that varied 10-fold in trichome density to examine their potential importance in the absorption of foliar-applied Zn. As stomata are also potentially important for the absorption of foliar-applied nutrients (Fernández *et al.*, 2013), we determined the densities of both the trichomes and stomata for leaves of the four NILs. As expected, trichome density differed markedly, with Pd1 (dense trichomes) having a density (~1100 cm^−2^) significantly higher than the other three NILs. Although Ps (sparse trichomes) had the lowest density (~100 cm^−2^), it was not significantly different to either P1 (glabrous; ~220 cm^−2^) or CS (wild-type; ~190 cm^−2^) (Fig. 6A, C). The trichomes on leaves of Pd1, CS, and Ps were similar to those described above in cv. Bunya (Fig. 1), with all three having ~95% Type V and ~5% Type IX. In contrast, P1 had shorter Type V trichomes compared to the other three NILs, with 35% being Type IX and the remainder being Type V (Fig. 6). The stomatal density was ~8000–10000 cm^−2^ for all four NILs, with no significant differences between them (Fig. 6B).

**Fig. 7.**
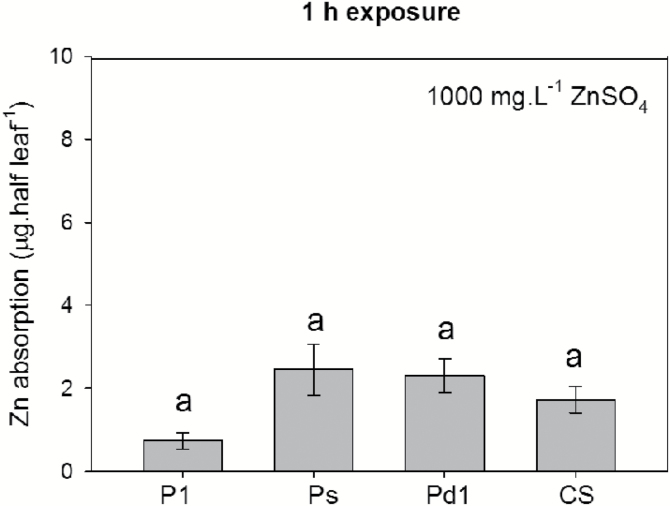
Comparison of the foliar absorption of Zn after exposure for 1 h to 1000 mg l^−1^ ZnSO_4_. Four near isogenic lines (NILs) of soybean that differed in trichome density were compared: P1 (L62-1385), Ps (L63-2999), Pd1 (L62-1686), and CS (L58-231). Data are means (±SD) (*n*=6). No significant differences were found among the four NILs (*P*<0.05). Foliar absorption of 1000 mg l^−1^ nano- and bulk-ZnO, and 3 mg l^−1^ ZnSO_4_ are not presented as the absorption was too low to be detected by ICP-MS.

**Fig. 8.**
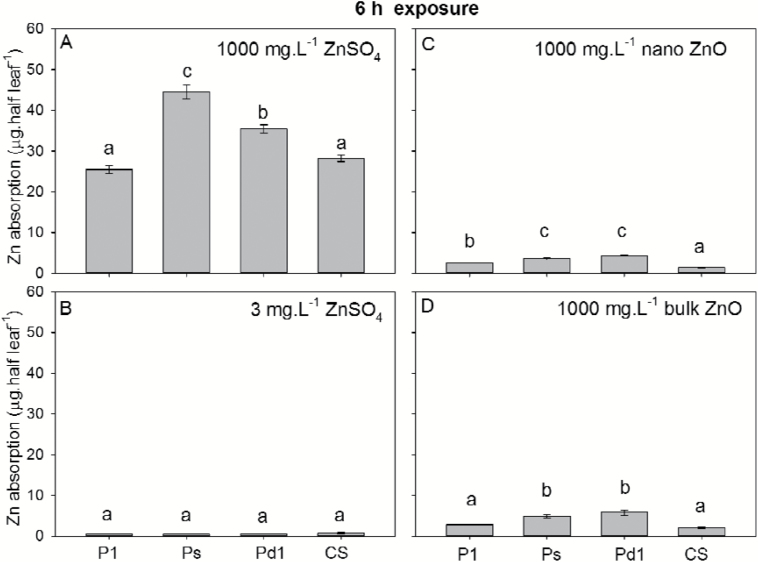
Comparison of the foliar absorption of Zn after exposure for 6 h to (A) 1000 mg l^−1^ ZnSO_4_, (B) 3 mg l^−1^ ZnSO_4_, (C) 1000 mg l^−1^ nano-ZnO, and (D) 1000 mg l^−1^ bulk-ZnO. Four near isogenic lines (NILs) of soybean were compared that differed in trichome density: P1 (L62-1385), Ps (L63-2999), Pd1 (L62-1686), and CS (L58-231). Data are means (±SD) (*n*=6). Different letters indicate significant differences (*P*<0.05).

Using ZnSO_4_, we then compared differences in the absorption of foliar-applied Zn between the four soybean NILs. A half-leaf loading method was used, with Zn applied to half the leaf and absorption calculated as the difference between the two halves of the leaf. After exposure for 1 h to 1000 mg l^−1^ ZnSO_4_, the quantity of Zn absorbed ranged from 0.7 to 2.5 μg per half leaf, with no significant differences found among the four NILs (Fig. 7). After exposure for 6 h, the amount of Zn absorbed increased to 25–45 μg per half leaf, and although significant differences were found between the four NILs, these were not related to trichome density. Indeed, comparing Pd1, CS, and Ps (all of which had the same proportion of trichome types), it was found that although Ps had the lowest trichome density (110 cm^−2^) it had the highest Zn absorption (45 μg per half leaf) (Figs 6, 8). In contrast, Pd1 (which had the highest trichome density among the three NILs, 1100 cm^−2^) had the second-highest Zn absorption (35 μg per half leaf) (Figs 6, 8).

### Absorption of Zn using a nano-fertilizer

Given the increasing interest in the use of nanoparticles as fertilizers, we also compared three types of Zn fertilizers, namely 1000 mg l^−1^ Zn supplied as either ZnSO_4_, nano-ZnO (~30 nm diameter), or bulk-ZnO (~200–400 nm diameter) (Supplementary Fig. S1). To facilitate comparison, the ZnSO_4_ was applied at two concentrations, 1000 and 3 mg l^−1^ Zn. The latter concentration was equal to the concentration of soluble Zn measured in suspensions of both 1000 mg l^−1^ nano- and bulk-ZnO. After 6 h, leaves exposed to 1000 mg l^−1^ ZnSO_4_ had the highest rates of Zn absorption (25–44 μg per half leaf), with absorption for leaves exposed to 1000 mg l^−1^ nano- or bulk-ZnO being about 10-fold lower (1–7 μg per half leaf) (Fig. 8). Interestingly, although the concentration of soluble Zn was the same in the nano- and bulk-ZnO treatments as it was in the 3 mg l^−1^ ZnSO_4_ treatment, the rate of absorption for leaves exposed to ZnSO_4_ was up to about 10-fold lower (0.5–0.7 μg per half leaf) than for leaves exposed to ZnO (Fig. 8). Across the different forms of Zn fertilizer, significant differences were found between the four NILs, but again these differences were not related to trichome density (Figs 6, 8).

## Discussion

Foliar fertilization is an agronomic strategy of increasing interest, particularly for micronutrients such as Zn where low concentrations within edible plant tissues also impact adversely upon human nutrition (Fernández *et al.*, 2013). However, despite their importance, the processes whereby foliar-applied nutrients move across the leaf surface remain unclear (Fernández *et al.*, 2017; Li *et al.*, 2017). Of particular interest, in the present study we investigated the role of trichomes in foliar absorption of Zn in soybean and tomato. Two types of trichomes were found on the leaf surface of soybean and seven types for tomato. Using synchrotron-based μ-XRF for *in situ* analyses of hydrated leaves after foliar Zn (ZnSO_4_) application, it was found that for soybean ~29% of Type V trichomes accumulated Zn at their base, but for tomato no trichomes were observed to accumulate Zn. Next, given that Zn was observed to accumulate in some trichomes on leaves of soybean, we examined this species in more detail by using NILs that differed up to 10-fold in trichome density, and also by using *in situ* analyses of Zn distribution in leaf cross-sections with μ-XRF. However, the results showed that trichomes were not a primary pathway for the foliar absorption of Zn in soybean either.

### Role of trichomes in absorption of foliar-applied Zn

We examined the distribution of Zn in leaves in order to determine if it accumulated in trichomes after foliar application. Using μ-XRF for *in situ* analysis of Zn distribution within hydrated leaves we found that, for both soybean and tomato, Zn moved rapidly across the leaf surface, and it was observed in the mesophyll within only 15 min of application (Fig. 3 and Supplementary Fig. S2). Zn accumulated first within the veins, with concentrations subsequently increasing in the interveinal tissues, especially in the apoplast (Fig. 4). Of particular interest, Zn accumulated at the base of ~29% of the non-glandular trichomes in soybean, especially within 3 h of application (Fig. 4 and Supplementary Fig. S3). This accumulation possibly occurred because the base of non-glandular trichomes may be less waxy and have a higher wettability compared to the cuticle (Brewer *et al.*, 1991; Grammatikopoulos and Manetas, 1994; Fernández *et al.*, 2014). Our findings were similar to the results of Schlegel and Schönherr (2002), who used Ag to visualize foliar uptake through the precipitation of AgCl in broad bean (*Vicia faba*) and maize (*Zea mays*). However, analysis of leaf cross-sections by µ-XRF showed that the Zn concentration was not higher in the cells surrounding the base of the trichomes (Fig. 5). Interestingly, we did not observe strong binding of Zn to the epidermal cells (i.e. Zn was moved into the underlying mesophyll cells) (Fig. 5), which has been proposed to account for the limited translocation of some foliar-absorbed nutrients, including Ca, Mn, Fe, and Zn (Zhang and Brown, 1999; Du *et al.*, 2015; Li *et al.*, 2017). Instead, we found that the Zn moved further into the leaf tissues (into the mesophyll cells) after only 15 min (Fig. 5).

To further quantify the overall importance of trichomes in foliar Zn absorption, we compared the absorption of foliar-applied Zn in four soybean NILs that differed up to 10-fold in their trichome density (Fig. 6). When supplied as a 1000 mg l^−1^ ZnSO_4_, it was found that after 1 h of exposure, although Zn tissue concentrations increased relative to the control, there were no significant differences between the four NILs despite their differing trichome densities (Fig. 7). Furthermore, although significant differences were found between the NILs after exposure for 6 h, they were not related to leaf trichome densities (Figs 6, 8). Thus, although Zn was found to accumulate at the base of some trichomes after foliar application (Fig. 4), they were not the primary organ for the movement of Zn across the leaf surface (Figs 6, 7, 8).

Our observation that trichomes do not appear to be the primary pathway for the movement of Zn across the leaf surface in either soybean or tomato (Figs 7, 8) is consistent with that of Burrows *et al.* (2013), who demonstrated that trichomes on the leaves of silverleaf nightshade (*Solanum elaeagnifolium*) did not facilitate the uptake of a fluorescent tracer. However, in contrast, it is known that peltate trichomes in the highly drought-resistant Bromeliaceae are associated with foliar absorption of water, Ca, and Zn (Ohrui *et al.*, 2007; Winkler and Zotz, 2010). Similarly, trichomes in a drought-tolerant cactus are involved in water collection from fog (Ju *et al.*, 2012), and trichomes in drought-stressed *Croton* can facilitate water penetration into leaves (Vitarelli *et al.*, 2016). It has also been reported that trichomes of temperate woodland ferns under high air humidity may have a role in foliar water uptake (Schwerbrock and Leuschner, 2017). Therefore, it seems that the role of trichomes differs widely, depending upon the plant species and the climatic and ecological conditions (Werker, 2000). Indeed, although it has been reported that the peltate trichomes of some Bromeliaceae species under drought conditions can absorb water and nutrients, which is helpful to overcome the dry conditions (Ohrui *et al.*, 2007; Winkler and Zotz, 2010), the stellate or peltate trichomes of some other terrestrial Bromeliaceae species are water repellant, which could potentially obstruct pathogens and aid in self-cleaning properties (Pierce *et al.*, 2001). Much remains unknown regarding the physico-chemical and ecological significance of trichomes (Bickford, 2016) and thus, although we found that they are not important for foliar absorption of Zn in soybean and tomato, it is possible that they could play a role in foliar absorption in other plant species. In addition, temperature, humidity, and the point of deliquescence of the nutrient compound also need to be considered when assessing foliar absorption (Kerstiens, 2006; Fernández *et al.*, 2017).

### Importance of other properties for absorption of foliar-applied Zn

Given that Zn absorption was not related to trichome density in the four soybean NILs, we also examined stomatal density; however, this did not differ between the four NILs (Fig. 6), and hence it could not account for the observed changes in foliar absorption of Zn (Fig. 8). We therefore considered whether the differences in absorption of Zn between the four NILs could be due to differences in contact between the leaves and the droplets, given that trichomes are known to alter leaf surface wettability (Brewer *et al.*, 1991; Fernández *et al.*, 2014). However, we found that the contact angles did not differ between the four NILs (Supplementary Figs S4, S5). This is consistent with observations by Brewer *et al.* (1991), who found that leaf water repellence was related to trichome density, particularly when the density was >25 mm^−2^ (in contrast, the trichome density of the NILs in the present study was 1–11 mm^−2^). Thus, differences in Zn absorption in the NILs could not be attributed to differences in wetting angle in the present study. Instead, it is possible that the differences could have been caused by, for example, different cuticle properties, although this was not examined in the present study. In addition, it should be noted that the RH during the foliar application period in our present study was very high (98%). This perhaps increased the cuticular permeation in the present study, as it has previously been suggested that cuticular permeation increases with RH, particularly when it is >90% (Schonherr, 2001; Fernández and Eichert, 2009; Fernández *et al.*, 2017).

### Pattern of accumulation of Zn in leaf tissues

It was observed that foliar-applied Zn accumulated more rapidly in the veins than in the interveinal tissues for both soybean and tomato (Fig. 4). This is consistent with previous studies, including that of Du *et al.* (2015), who reported that foliar-applied Zn accumulated in the highest concentrations in the veins of tomato and citrus (*Citrus reticulatus*). Similarly, Li *et al.* (2017) found that concentrations of foliar-absorbed Fe, Mn, and Zn in the veins were 2- to 4-fold higher than in the corresponding interveinal tissues in both sunflower (*Helianthus annuus*) and tomato. However, the reasons for this rapid accumulation in the veins remain unclear. Given that leaf wettability is an important factor influencing the absorption of foliar-applied nutrients (Fernández and Brown, 2013; Fernández *et al.*, 2013), we measured the contact angle of Zn droplets overlaying either veins or the interveinal tissues. We found that the contact angle of the droplets above veins was half that of droplets above interveinal tissues (Supplementary Fig. S6). Therefore, we suggest that the initial rapid accumulation of Zn in the vein (Fig. 4) potentially occurs due to the higher wettability of the vein compared to the interveinal tissues, thereby resulting in better adhesion and contact.

### Effect of the form of Zn on foliar absorption

It has been proposed that nano-based foliar fertilizers may be more effective than traditional ones. For example, it is possible to alter the surface functionalization of nano-based fertilizer products, such as for preparing fertilizers with sustained release rates (DeRosa *et al.*, 2010). It has previously been shown that nano-fertilizers can be absorbed by both the leaves and roots (Slomberg and Schoenfisch, 2012; Hussain *et al.*, 2013), but it is unclear as to whether it is the nanoparticles themselves that are taken up or their dissolution products (Wang *et al.*, 2016, 2017). In the present study, we examined nano-ZnO (~30 nm diameter) and homologous bulk-ZnO (~200–400 nm diameter) (Supplementary Fig. S1). For an additional control, we also examined ZnSO_4_ at a Zn concentration of 3 mg l^−1^, which corresponded to the same soluble Zn concentration measured for the nano- and bulk-ZnO suspensions. We exposed the leaves of the four soybean NILs to the compounds for 6 h, and found that absorption for 1000 mg l^−1^ ZnSO_4_ was on average about 10-fold higher than for the nano- and bulk-ZnO suspensions. Moreover, there were no marked differences in foliar absorption between the nano- and bulk-ZnO particles, which differed 10-fold in their particle size. The markedly lower absorption upon exposure to the nano- and bulk-ZnO suspensions compared to 1000 mg l^−1^ ZnSO_4_ coupled with the similar absorption for nano- and bulk-ZnO, suggests that the movement of Zn across the leaf surface mainly occurs as soluble Zn, rather than as solid ZnO. This is perhaps surprising, given that the nano-ZnO had an average particle diameter of 30 nm, which is smaller than typical stomatal openings (approx. 2 × 9 µm) (Supplementary Fig. S7). It therefore appears that ZnO was not greatly absorbed through the stomata, although the general role of stomata in foliar absorption remains unclear (Fernández and Brown, 2013). Nevertheless, it is possible that the slower absorption of Zn from the nano- and bulk-ZnO suspensions could actually be advantageous, not only in avoiding the ‘burning’ that can occur with soluble forms (Drissi *et al.*, 2015), but also in providing a prolonged source of Zn (i.e. a slow-release fertilizer) if these ZnO particles can be retained on the leaf surface. Further research in this regard would clearly be valuable.

Interestingly, the extent of absorption upon exposure to 1000 mg l^−1^ nano- and bulk-ZnO suspensions was on average about 6-fold higher than when exposed to 3 mg l^−1^ ZnSO_4_. This difference occurred despite the concentration of soluble Zn (i.e. 3 mg l^−1^) being the same across the three treatments. It is possible that the foliar absorption of soluble Zn from the ZnO suspensions on the leaf surface caused the continual dissolution of the remaining ZnO, and hence increased absorption compared to the 3 mg l^−1^ ZnSO_4_ treatment.

The point of deliquescence of different compounds can influence their rate of foliar absorption (Fernández and Eichert, 2009; Fernández *et al.*, 2017), although this is mainly important in longer-term experiments, such as those where droplets have sufficient time to potentially dry out. In our present study, however, droplets were applied for ≤6 h and remained as liquid throughout. Given that the absorption differed by 10-fold between the three Zn compounds, this cannot be attributed to differences in the point of deliquescence. Instead, it was due to differences in the soluble Zn concentration.

## Conclusions

Despite the importance of foliar fertilization for improving crop nutrition, the mechanisms by which foliar-applied nutrients move across the leaf surface remain unclear, including the potential role of trichomes. Using synchrotron-based μ-XRF and NILs of soybean differing up to 10-fold in trichome density, we found that trichomes are not part of the primary pathway by which foliar-applied Zn moves across the leaf surface in soybean and tomato. However, it is important this work should be extended to other plant species that are known to differ in their leaf properties and trichome types. In addition, we also compared soluble Zn applied as ZnSO_4_ with sparingly soluble Zn as nano- and bulk- ZnO, and found that it was mainly absorbed as the soluble form (irrespective of whether ZnO was supplied in nano or bulk forms). Zn absorption upon exposure to ZnSO_4_ was about 10-fold higher than for either nano- or bulk-ZnO after 6 h of application. However, we observed continued absorption of soluble Zn from suspensions of nano- and bulk-ZnO, which appeared to result in their gradual dissolution. This suggests that they may potentially be useful as slow-release fertilizers, provided that they can be retained on the leaf surface. Further studies are required in this regard.

## Supplementary data

Supplementary data are available at *JXB* online.

Fig. S1. Scanning electron micrographs showing particles of the nano- and bulk-ZnO.

Fig. S2. Tomato µ-XRF survey scans.

Fig. S3. Tri-color image of a soybean leaf scanned from underneath where a ZnSO_4_ droplet had been applied for 1 h.

Fig. S4. Comparison of the contact angles of the three Zn fertilizers on the leaves of the four soybean near-isogenic lines.

Fig. S5. Light micrographs showing contact angles of droplets on the four soybean near-isogenic lines.

Fig. S6. Images showing droplets applied either over a vein or over interveinal tissue of a soybean leaf.

Fig. S7. Scanning electron micrograph showing the size of the opened stomata of a soybean leaf.

Table S1. Results from dynamic light-scattering of the nano- and bulk-ZnO compounds.

Supplementary Figures and Tables

## Abbreviations

NILs: near-isogenic lines
ICP-MS: inductively coupled plasma mass spectrometry
μ-XRF: synchrotron-based X-ray fluorescence microscopy
YFELs: youngest fully expanded leaves
RH: relative humidity
