# Supplementary material for: Absorption of foliar-applied Zn fertilizers by trichomes in soybean and tomato
Source: J Exp Bot. 2018 Mar 3;69(10):2717–29. doi: 10.1093/jxb/ery085 (PMC5920297; doi:10.1093/jxb/ery085)
Supplement: Supplementary Figures and Tables [file ery085_suppl_supplementary_figures_and_tables.pdf]

SUPPLEMENTARY DATA

Absorption of foliar applied Zn fertilizers by trichomes in soybean and tomato

Cui Li[1], Peng Wang[1, 2, *], Enzo Lombi[3], Miaomiao Cheng[4], Caixian Tang[4], Daryl L. Howard[5], Neal W. Menzies[1], Peter M. Kopittke[1]

[1] The University of Queensland, School of Agriculture and Food Sciences, St Lucia, Queensland, 4072, Australia

[2] Nanjing Agricultural University, College of Resources and Environmental Sciences, Nanjing, 210095, China

[3] University of South Australia, Future Industries Institute, Mawson Lakes, South Australia, 5095, Australia

[4] La Trobe University, Centre for AgriBioscience, Bundoora, Victoria, 3086, Australia

[5] ANSTO, Australian Synchrotron, Clayton, Victoria, 3168, Australia

Author for correspondence:
Peng Wang
Tel: +86 25 84399055, Fax: 86 25 84399055
Email: p.wang3@njau.edu.cn

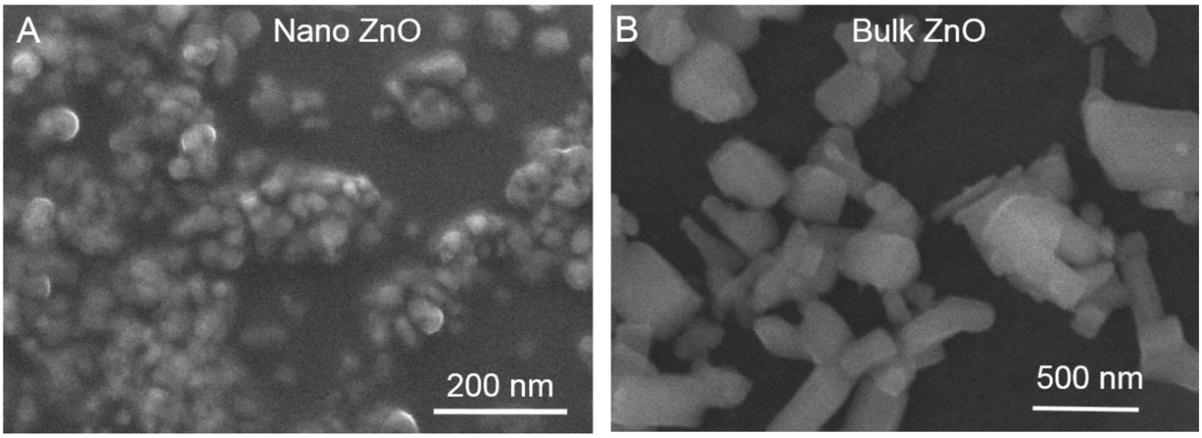

Fig. S1 Scanning electron micrographs showing particles of the nano-ZnO (A) and the bulk-ZnO (B).

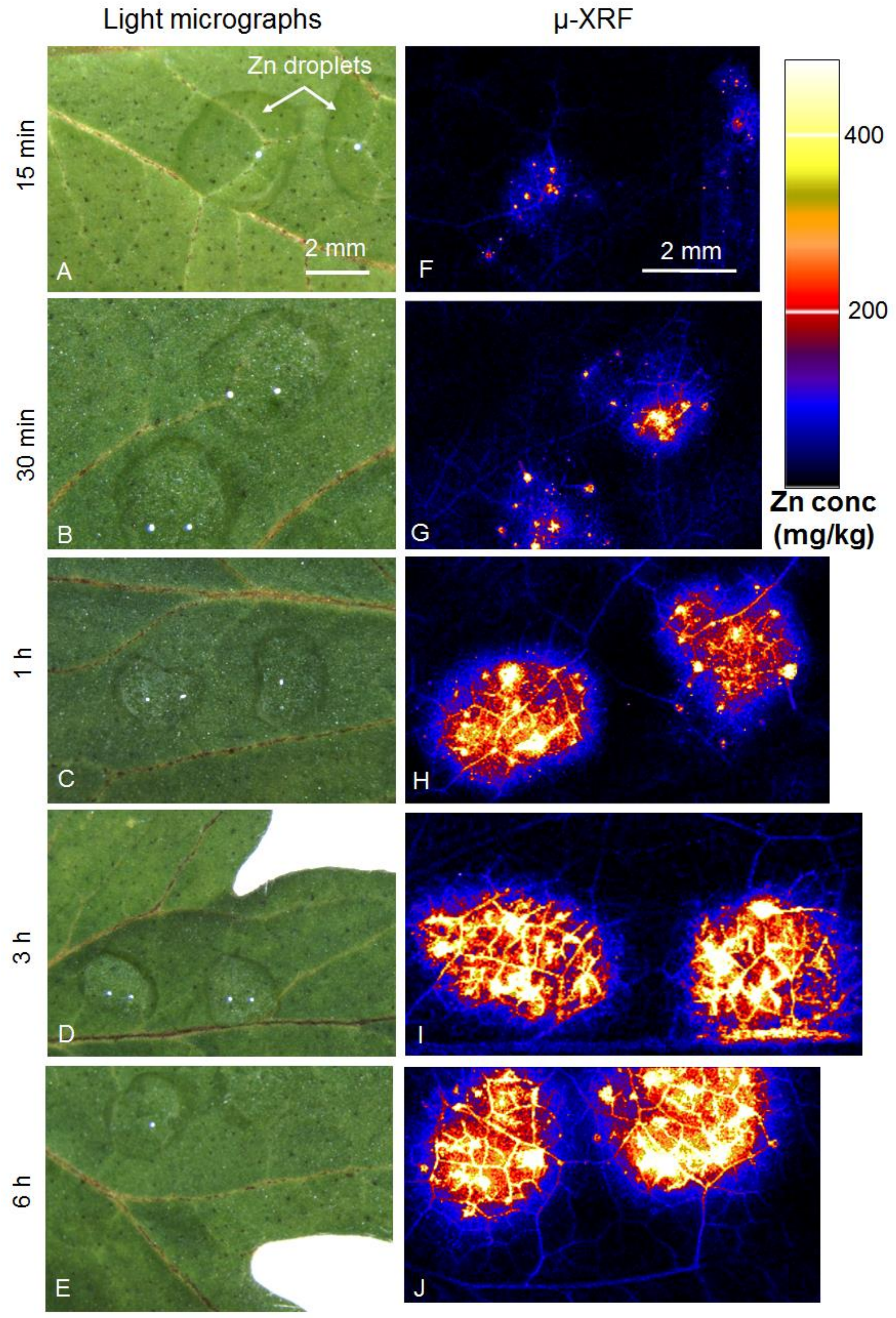

Fig. S2 (A-E) Light micrographs showing the Zn droplets ($ZnSO_4$) on tomato leaves before being removed and rinsed. (F-J) Results from the µ-XRF survey scans showing the distribution of Zn. The images in (F-J) have the same color scale, with brighter colors corresponding to higher Zn concentrations (mg/kg). Droplets were left on the leaf surface for 15 min, 30 min, 1 h, 3 h, or 6 h. The scale bar in (A) applies to (A-E), and the scale bar in (F) applies to (F-J).

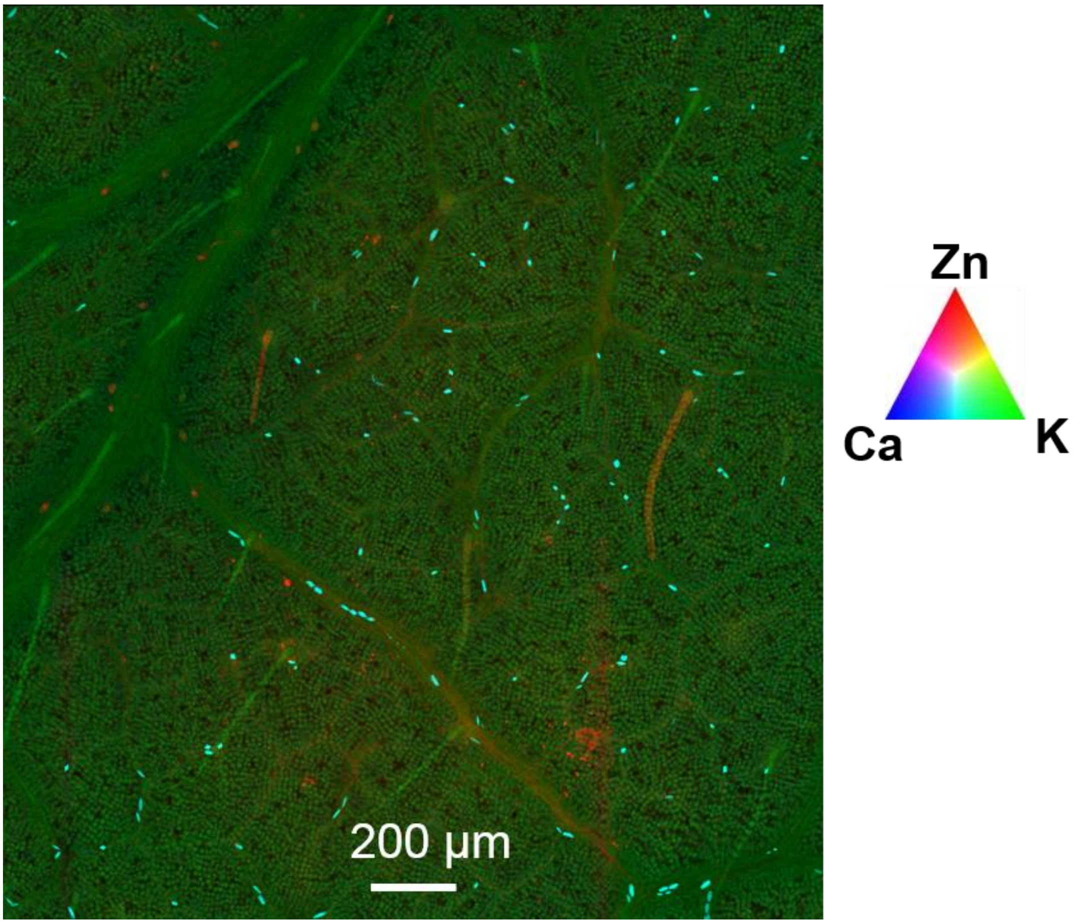

Fig. S3 Tri-color image (detailed scan; red is Zn, K is green, and Ca is blue) of a soybean leaf scanned from underneath where a $ZnSO_4$ droplet had been applied for 1 h. Many Type V trichomes can be seen (ca. 200-400 µm long), with some containing elevated concentrations of Zn.

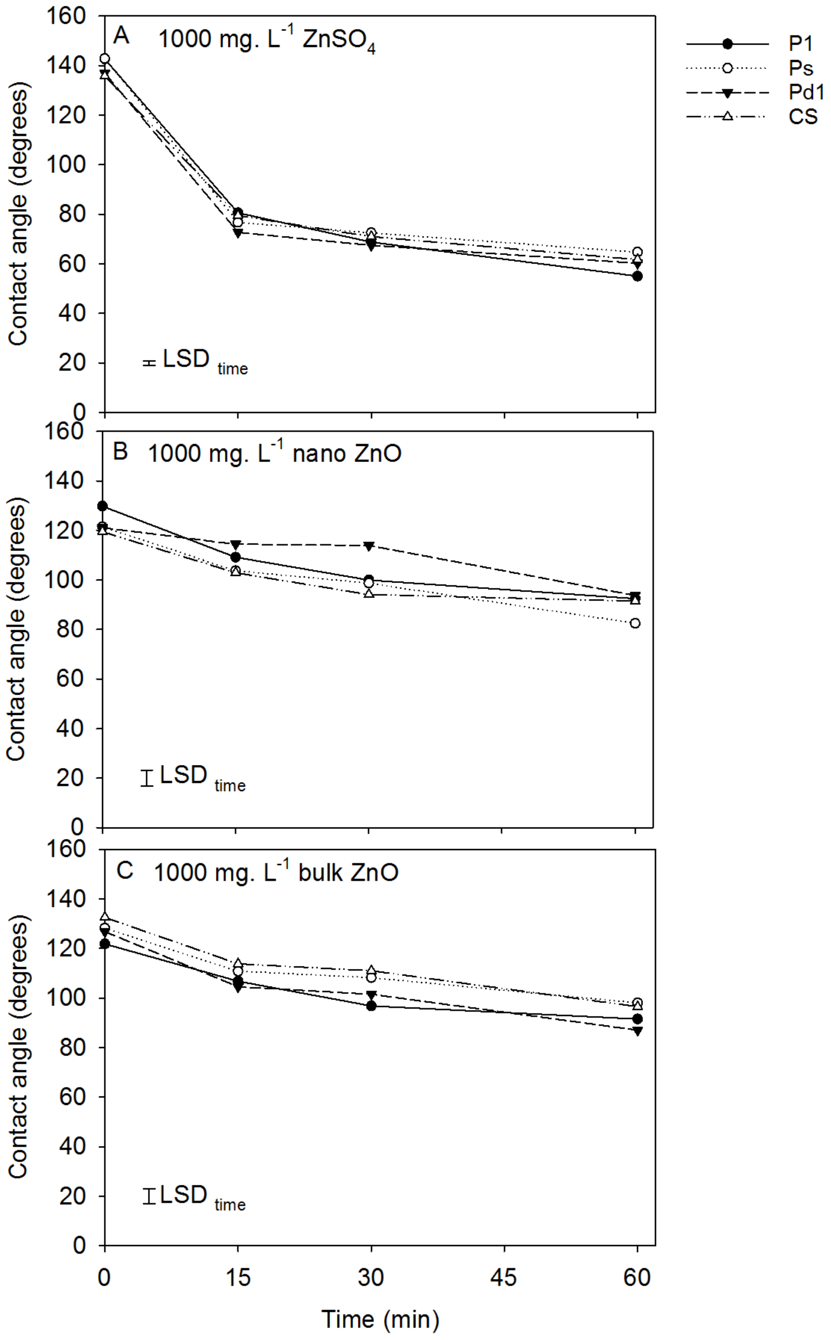

Fig. S4 Contact angles for 5 µL droplets of 1000 mg L^{-1} $ZnSO_4$ (a), 1000 mg L^{-1} nano-ZnO (b), and 1000 mg L^{-1} bulk-ZnO (c) droplets (all solutions with 0.05 % Tween 20). The droplets were applied to leaves of four near-isogenic lines (NILs) of soybean. Measurments were taken 0, 15, 30, and 60 min after applying the droplets (n = 6). In all instances, for the two-way analysis of variance there was no significant interaction (NIL and time) or any significant differences between NILs. Therefore, the only LSD values (5 %) shown are for time, with contact angles generally decreasing over time.

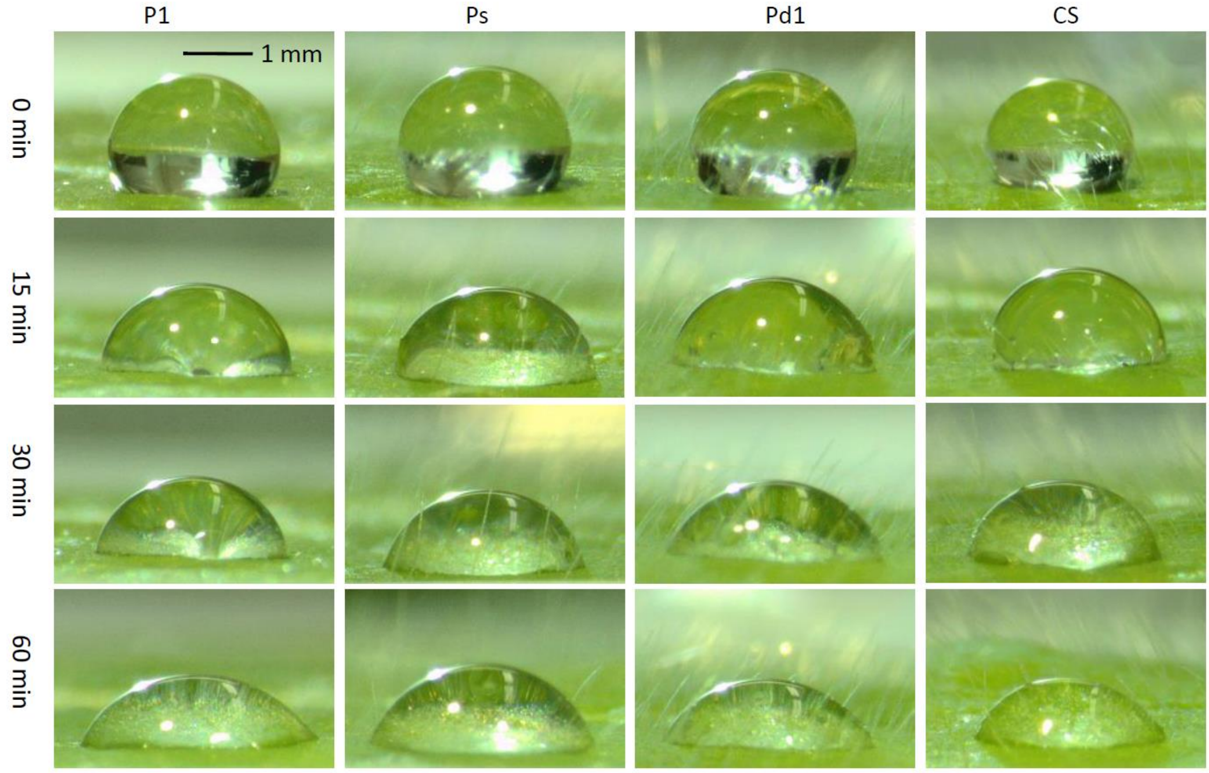

Fig. S5 Light micrographs from which the contact angles were calculated for four near isogenic lines (NILs) of soybean. Droplets (5 µL) were applied to interveinal leaf tissues using 1000 mg L^{-1} $ZnSO_4$, with images captured after 0, 15, 30, and 60 min. All droplets contained 0.05 % Tween 20. Only data for 1000 mg L^{-1} $ZnSO_4$ are shown here, with the images for nano-ZnO and bulk-ZnO being similar.

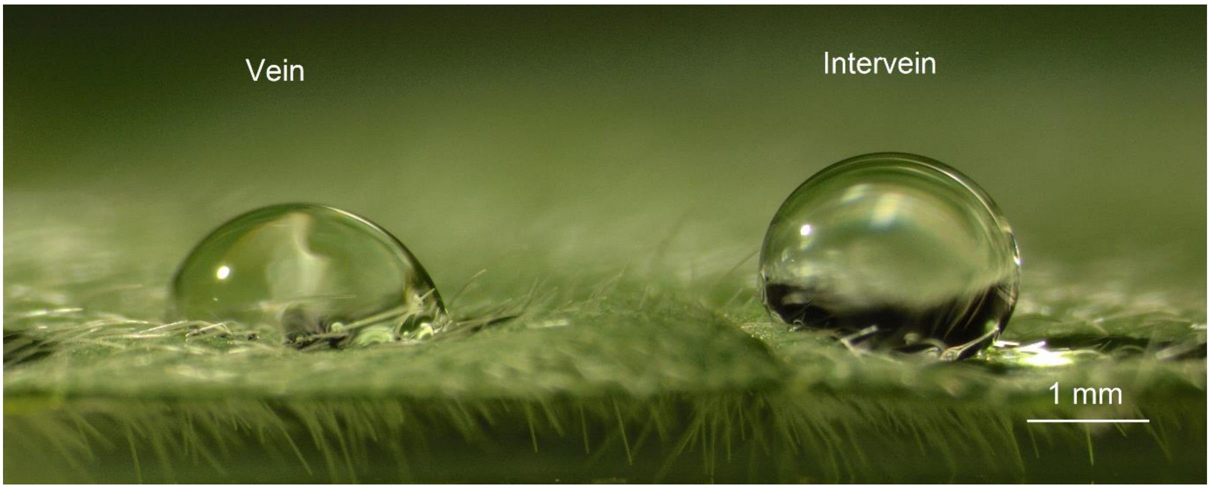

Fig. S6 Comparison of the contact of droplets of 1000 mg L^{-1} $ZnSO_4$ (5 µL droplets with 0.05 % Tween 20) with the vein and the interveinal tissue of a soybean leaf. The image was captured immediately after the droplets had been applied.

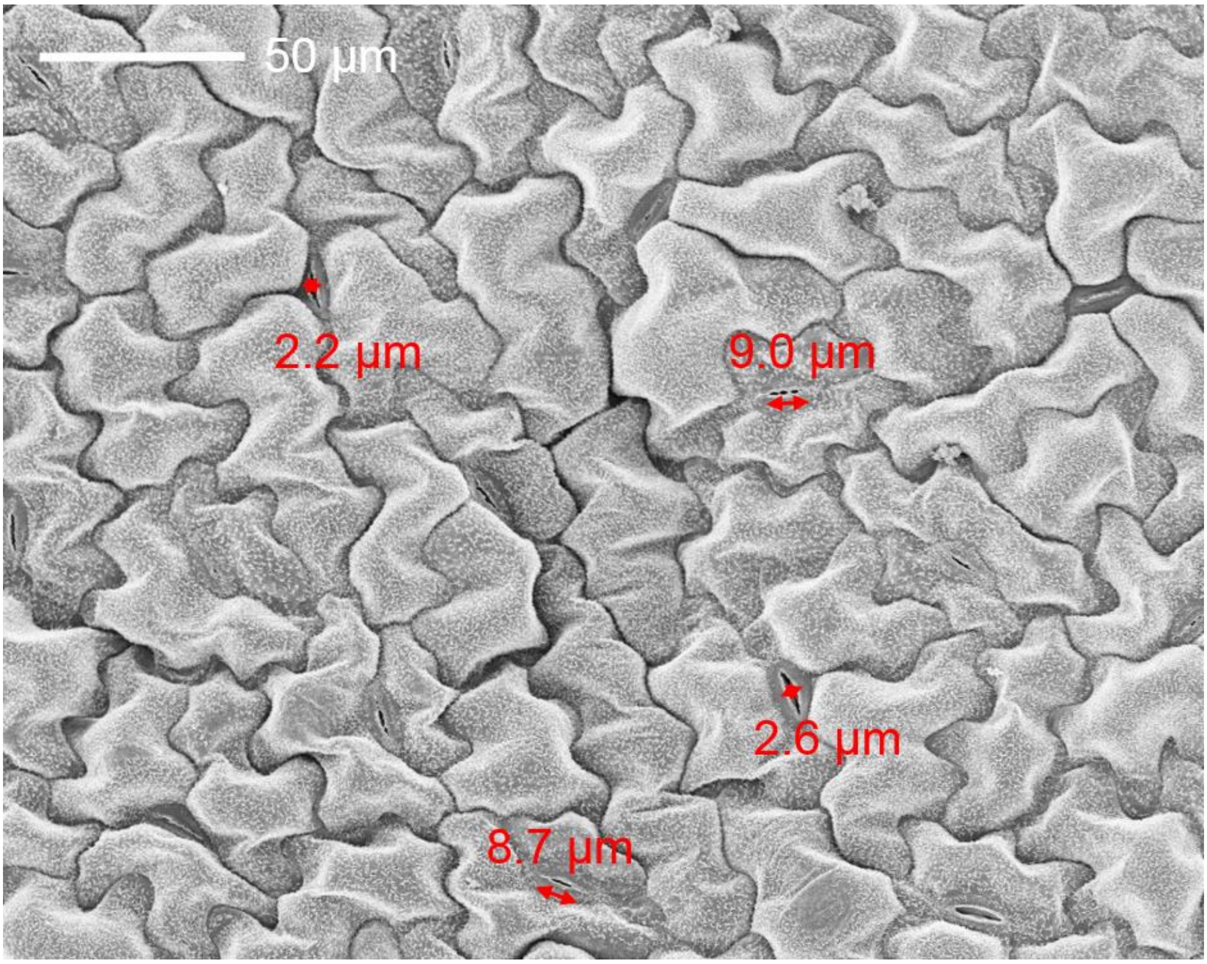

Fig. S7 Scanning electron micrograph showing opening stomatal size of P1 L62-1385 (one of the four soybean near-isogenic lines, with the other three having similar stomatal size).

Table S1 Results of analyses using dynamic light scattering (DLS) for the nano- and bulk-ZnO. The numbers in brackets are the relative abundance of particles of each size (%).

Product	Fit Error	Residual	Mean diameter (nm)			
			Volume		Number	
			Peak 1	Peak 2	Peak 1	Peak 2
Bulk-ZnO	2.13	5.52	154 (15.9)	555 (84.1)	143 (89.7)	506 (10.3)
Nano-ZnO	2.59	0.00	59.4 (72.1)	173 (27.9)	55.9 (98.4)	161 (1.60)
